# Draft Genome Sequence of *Pseudomonas mosselii* Gil3, Isolated from Catfish and Antagonistic against Hypervirulent *Aeromonas hydrophila*

**DOI:** 10.1128/genomeA.01305-16

**Published:** 2016-11-17

**Authors:** Dunhua Zhang, De-Hai Xu, Junqiang Qiu, Cody R. Rasmussen-Ivey, Mark R. Liles, Benjamin H. Beck

**Affiliations:** a Aquatic Animal Health Research Unit, USDA-ARS, Auburn, Alabama, USA; b College of Fisheries and Life Sciences, Shanghai Ocean University, Pudong New District, Shanghai, China; c Department of Biological Sciences, Auburn University, Auburn, Alabama, USA

## Abstract

*Pseudomonas mosselii* Gil3 was isolated from a catfish that survived from lethal challenge with hypervirulent *Aeromonas hydrophila* (vAh). When assayed *in vitro*, the bacterium showed antagonism against vAh. Sequence analysis revealed that the genome of *P. mosselii* Gil3 encodes numerous aromatic metabolism pathways and proteins for biosynthesis of antimicrobial compounds.

## GENOME ANNOUNCEMENT

Environmental bacteria within the *Pseudomonas putida* group (1) colonize diverse terrestrial and aquatic habitats and are of interest for their biodegradation properties (2), their antagonistic secondary metabolites (3), and their potential in biotechnological applications (4, 5). A *P. putida*–like strain, Gil3, was isolated from the gill tissue of a channel catfish (*Ictalurus punctatus*) that survived from a lethal challenge of hypervirulent *Aeromonas hydrophila* (vAh) (6). This strain showed an antagonistic effect against vAh in *in-vitro* assays and was initially identified as *P. putida* Gil3 based on 16S rRNA homology to *P. putida* BW11M1 (7) and *Pseudomonas* sp. 250J (8), both of the latter being recognized as antibiotic-producing soil/rhizosphere isolates. The identity of this strain was reclassified as *P. mosselii* Gil3 (also *P. putida* BW11M1 as *P. mossselii* BW11M1) when the calculated value of average nucleotide identity was compared with that of the type species within the *P. putida* group. Since vAh is one of the most important bacterial pathogens that causes persistent outbreaks of motile *Aeromonas* septicemia (MAS) in warm-water fishes (9, 10), approaches are sought to suppress the pathogen and control the MAS disease. To explore the potential of *P. mosselii* Gil3 as a probiotic against vAh, the genome of the strain was sequenced in this study.

Genome sequencing was performed on the Illumina MiSeq sequencer using a Nextera XT kit (Illumina, San Diego, CA, USA) and a 2 × 250-bp paired-end sequencing kit. Resulting sequences were trimmed with a quality cutoff of 0.01 using CLC Genomics Workbench (CLCBio, Cambridge, MA, USA). A total of 101 contigs were *de novo* generated using the SPAdes Genome Assembler version 3.5.0 with an average coverage of 43.6-fold. The average contig size was 57,550 bp with an *N*_50_ value of 135,596 bp. The assembled genome size was 5,812,302 bp with a G+C content of 64.2%. The draft genome was annotated using RASTtk (http://rast.nmpdr.org) and the NCBI Prokaryotic Genome Annotation Pipeline (https://www.ncbi.nlm.nih.gov/genome/annotation_prok). A total of 5,286 coding genes were identified, including four copies of 5S rRNA, two copies of 16S rRNA, and one copy of 23S rRNA. Among coding genes, 5,207 coding sequences were associated with 470 metabolic subsystems.

Several aromatic metabolism pathways were identified in *P. mosselii* Gil3, including salicylate and gentisate catabolic pathways, a hydroxyphenylacetic acid catabolic pathway and a β-ketoadipate pathway. Additionally, *P. mosselii* Gil3 contains gene clusters predicted for the synthesis of antimicrobial compound xantholysin (11) and a *ppyS* homologous gene for synthesis of antibiotic pseudopyronines (12). Phylogenetic analysis of concatenated multilocus genes (*gyrB*, *recA*, *ropB*, *ropD*, and *NuoD*) showed that *P. mosselii* Gil3 forms a clade with 100% bootstrap support with *Pseudomonas* sp. 250J (accession no. JHEE00000000), *P. putida* 1A00316 (CP014343), *P. mosselii* DSM 17497 (JHYW00000000), and *P. mosselii* BW11M1 (LSLE00000000).

### Accession number(s).

This whole-genome shotgun project has been deposited in DDBJ/ENA/GenBank under the accession number LZCV00000000. The version described in this paper is the first version, LZCV01000000.
